# Academic Primer Series: Five Key Papers about Study Designs in Medical Education

**DOI:** 10.5811/westjem.2017.4.33906

**Published:** 2017-05-15

**Authors:** Michael Gottlieb, Teresa M. Chan, Jenna Fredette, Anne Messman, Daniel W. Robinson, Robert Cooney, Megan Boysen-Osborn, Jonathan Sherbino

**Affiliations:** * Rush University Medical Center, Department of Emergency Medicine, Chicago, Illinois; † McMaster University, Department of Medicine, Division of Emergency Medicine, Hamilton, Ontario, Canada; ‡ Christiana Care Health System, Department of Emergency Medicine, Newark, Delaware; § Wayne State University, Department of Emergency Medicine, Detroit, Michigan; ¶ University of Chicago, Department of Medicine, Division of Emergency Medicine, Chicago, Illinois; || Geisinger Medical Center, Department of Emergency Medicine, Danville, Pennsylvania; # University of California, Irvine, Medical Center, Department of Emergency Medicine, Orange, California

## Abstract

**Introduction:**

A proper understanding of study design is essential to creating successful studies. This is also important when reading or peer reviewing publications. In this article, we aimed to identify and summarize key papers that would be helpful for faculty members interested in learning more about study design in medical education research.

**Methods:**

The online discussions of the 2016–2017 Academic Life in Emergency Medicine Faculty Incubator program included a robust and vigorous discussion about education study design, which highlighted a number of papers on that topic. We augmented this list of papers with further suggestions by expert mentors. Via this process, we created a list of 29 papers in total on the topic of medical education study design. After gathering these papers, our authorship group engaged in a modified Delphi approach to build consensus on the papers that were most valuable for the understanding of proper study design in medical education.

**Results:**

We selected the top five most highly rated papers on the topic domain of study design as determined by our study group. We subsequently summarized these papers with respect to their relevance to junior faculty members and to faculty developers.

**Conclusion:**

This article summarizes five key papers addressing study design in medical education with discussions and applications for junior faculty members and faculty developers. These papers provide a basis upon which junior faculty members might build for developing and analyzing studies.

## INTRODUCTION

A thorough understanding of study design is essential for creating successful studies.^1^ While there are multiple approaches to designing an experiment, one must understand the limitations inherent in each technique, as well as potential biases and challenges that may result from a selected approach. One must be thoughtful and cognizant of this prior to beginning a project, as errors in study design and data collection can severely compromise a study’s results. Additionally, it is important to understand these limitations when evaluating a study as a peer reviewer, as well as when applying and interpreting studies for clinical or educational use.

While the Accreditation Council for Graduate Medical Education (ACGME) requires residents to participate in research, the degree of involvement in the process and training can be variable.^2^ After completing residency, junior faculty members may start their careers without having had sufficient training or mentorship in study design for medical education.^3^,^4^ They may then struggle to successfully produce high-quality scholarship.

The Faculty Incubator was created by the Academic Life in Emergency Medicine (ALiEM) team to provide early-career educators with a community of practice where they can discuss and debate topics relevant to the 21st century medical educator. To that end, we created a one-month module focused on study design.

This paper is a narrative review that highlights some important literature that may assist junior educators seeking to learn more about study designs in medical education.

## METHODS

In the eighth month of the ALiEM Faculty Incubator (October 1–31, 2016), we discussed the topic of study design for medical education. We monitored the proceedings of this group of educators from October 1–31, 2016. Our online discussions involved both junior faculty members and faculty mentors. While discussions occurred, we gathered the titles of papers that were cited, shared, and recommended within our online discussion forum and compiled these into a list. We also asked all of the monthly mentors for additional suggestions on relevant literature.

Once the augmented list was completed, we then conducted a three-round voting process, inspired by the Delphi methodology similar to our previous papers, to build consensus on which papers to feature.^5^–^8^ The first round asked the group to rate the article on a scale of 1 to 7. The second round used the existing, blinded data from round 1 to determine whether the article should be included or not. The final round asked the group to select the top five articles for inclusion, with consensus determined by the top five papers receiving a clear majority of the voting. This was not a traditional Delphi methodology since our selection panel was comprised of both novices (i.e. junior faculty members, participants in the Faculty Incubator) and experts in the field (i.e., experienced clinician educators, all of whom have published >10 peer-reviewed medical education publications, who serve as mentors and facilitators of the ALiEM Faculty Incubator). However, we intentionally used this method to involve both junior and experienced clinician educators to ensure we selected papers that would be of use to a spectrum of educators throughout their careers. There were four novice and four experienced medical educators involved in the analysis. All eight members were emergency medicine specialists. All members participated in all rounds of voting with 100% response rates for all rounds.

## RESULTS

Our ALiEM Faculty Incubator discussions in combination with expert recommendations yielded a total of 29 articles. Our approach allowed us to create a rank-ordered listing of all of the papers in order of perceived relevance, from the most to the least relevant. The top five papers were expanded upon below. Our ratings of all 29 papers are listed in the table, along with their full citations.

## DISCUSSION

The following is a list of papers that we determined to be of interest and relevance to junior faculty members and faculty developers. The accompanying commentaries explain the relevance of these papers to junior faculty members, while highlighting considerations for senior faculty members when using these publications for faculty development workshops or sessions.

### 1. Bordage G and Dawson B. Experimental study design and grant writing in eight steps and 28 questions. *Med Educ*. 2003;37(4):376–85.^9^

#### Summary

Creating a research question, designing a study, and writing a grant proposal are important skills for the physician educator-researcher. This article provides an eight-step, 28-question guide for researchers to follow at the beginning of the design process to ensure that all elements of design have been carefully considered. The guide incorporates the author’s prior work, explaining common reasons why manuscripts are accepted or rejected from medical education journals.^10^ It examines how to define a relevant research question, study design and appropriate statistics, the importance of sample size and sampling procedure, budget and personnel requirements, and writing grant proposals. While this process is best applied to experimental studies the principles outlined are applicable to a wide array of other research designs.

#### Relevance to Junior Faculty Members

It can be difficult for a novice researcher to choose an appropriate research question and properly design a study. Using this 28-question approach, this paper may provide guidance to junior faculty members who are planning research studies. By considering these important design questions, junior faculty may improve the strength of their research, produce more meaningful outcomes, and have better publication success.

#### Considerations for Faculty Developers

Faculty developers may find this paper to be a valuable resource for junior faculty members as they become more involved in research and grant writing. The list provides a more manageable approach to research, allowing the faculty developer to expand upon this with both experiential examples and further directions. This could also be used as pre-reading for a research course or as a resource for mentees.

### 2. Crites GE, Gaines JK, Cottrell S, et al. Medical education scholarship: an introductory guide: AMEE Guide No. 89. *Med Teach*. 2014;36(8):657–74.^11^

#### Summary

Faculty members who wish to advance their careers must produce scholarship. This article provides guidance for planning a scholarly project and advancing one’s career. It begins with a brief overview of the different types of scholarship with particular emphasis on the scholarships of discovery and teaching.^12^ Next, the authors provide specific advice in the planning of a scholarly project. This advice includes best practices on finding a mentor. Then, the reader is advised to set clear goals with particular guidance provided on how to develop a good research question, as well as a seven-step scholarship plan. The authors recommend the use of educational theories or conceptual frameworks to guide the scholarly plan. The authors also provide advice on which particular research methods to employ, depending on the type of scholarship the reader is attempting to produce. The final steps that the authors recommend are for the reader to determine whether their scholarly project is adequate and, if so, how to present the results of the scholarly project. The authors emphasize throughout the article the importance of understanding one’s promotion and tenure requirements at one’s institution.

#### Relevance to Junior Faculty Members

This paper is a must-read for junior faculty members. It provides invaluable advice regarding creation of a scholarly project, as well as general advice for junior faculty members to help advance their career. There is specific advice on the importance of obtaining a mentor and how to be a good mentee. Most importantly, the paper is well-referenced so that if the reader has further questions regarding a particular topic, finding further information is very easy.

#### Considerations for Faculty Developers

This paper provides valuable tips for faculty developers on how to be effective mentors, as well as advice to provide mentees on establishing and maintaining successful relationships. Additionally, this can serve as a blueprint for how to advise junior faculty on the creation of scholarly projects, emphasizing the role of the mentor at each step.

### 3. Yarris LM, Deiorio NM. Education research: a primer for educators in emergency medicine. *Acad Emerg Med*. 2011;18 Suppl 2:S27–35.^13^

#### Summary

Yarris and Deiorio provide a nice overview of education research for more-novice researchers. They provide a sequential approach to research, beginning with formulating appropriate and testable study questions. They emphasize the importance of performing a thorough literature review and using the *FINER* (feasible, interesting, novel, ethical, and relevant) approach to developing the research question. The authors subsequently provide a brief review of the various study designs, giving equal weight to both quantitative and qualitative approaches. Finally, the authors provide an approach to dissemination, as well as an extensive list of potential journals dedicated to reporting research in medical education. Throughout the paper, the authors provide numerous examples, as well as approaches to overcoming barriers with each step along the research pathway.

#### Relevance to Junior Faculty Members

This article provides a valuable overview of the research process within medical education for more-novice researchers. Given the importance of selecting appropriate and testable hypotheses, junior faculty may find the sections on question design particularly valuable to ensure that the study concept is feasible and likely to be useful to the broader community. Additionally, the discussion of different approaches to study design can help with understanding limitations and the best approach to testing one’s study question. Importantly, this paper discusses both quantitative and qualitative research methodology, explaining the differences between the approaches and how each could be applied to study design. Qualitative research is particularly valuable within medical education research yet is poorly taught in comparison with traditional, clinical research. In the latter portion of the article, the authors provide lists of potential funding sources, as well as outlets for dissemination of medical education scholarship, which can also be invaluable resources for junior faculty.

#### Considerations for Faculty Developers

Completion of scholarly activity by faculty is the most frequently encountered cause for a cautionary ACGME citation when emergency medicine residency programs undergo reviews.^14^ For this reason, it is imperative that faculty focus on the completion of scholarly activity. Despite the teaching inherent in a faculty role, these educators may not be aware that certain products of teaching can be considered scholarship. This paper provides a simple primer that faculty developers may use to guide faculty to begin generating educational scholarship. The primer covers various formats used within educational scholarship. While brief, this overview is valuable for guiding faculty in the beginning phase of their scholarship. The article concludes with a comprehensive list of journals that accept educational scholarship to help faculty disseminate scholarly products. When combined with the work on the scholarship of teaching by Glassick,^15^ this article provides a foundation for faculty to get credit for more than simply teaching.

### 4. Ramani S, Mann K. Introducing medical educators to qualitative study design: twelve tips from inception to completion. *Med Teach*. 2016;38(5):456–63.^16^

#### Summary

Ramani and Mann provide a focused introduction to qualitative research in medical education. They simplify qualitative research into 12 steps to help guide the novice researcher. Initially, the authors set the groundwork for understanding how qualitative research is relevant to medical education given some of the skepticism about qualitative research. However, medical educators and clinicians are becoming increasingly accepting of qualitative research and the rigor it requires. The authors suggest the following 12 steps: 1) choose a framework (e.g. ethnography, phenomenology, grounded theory, or discourse analysis); 2) understand reflexivity in that the researcher and methods influence the data; 3) understand how to mitigate ethical concerns; 4) know how to sample the population; 5) match the source data to the framework and the intended study outcome; 6) understand how to perform data collection; 7) prepare the data for analysis; 8) analyze the initial data; 9) determine if initial analysis is necessary and resolve internal team thematic conflicts; 10) maintain rigor; 11) report the results; and 12) be aware that specific training in qualitative methods is often necessary.

#### Relevance to Junior Faculty Members

Understanding how and why to do qualitative research is often a daunting task for the novice researcher who may not have received formal training in these research methods. This article breaks this approach into reasonable steps. As each of the 12 steps requires a more in-depth understanding than one article can provide, this paper serves as a nice initial framework for understanding qualitative methods. Junior faculty members interested in performing qualitative research are advised to expand upon this, using additional resources including many of the publications cited in this article.

#### Considerations for Faculty Developers

Qualitative methodology has taken the medical education field by storm in the past decade. Thus, any medical education interest group or journal club will undoubtedly fold qualitative research into their proceedings. Most junior faculty come from biomedical backgrounds, however, and may find these techniques quite foreign. It is therefore incumbent upon faculty development leaders to provide guidance and teaching centered on these types of research methods. Although this paper will not make a new junior faculty member immediately adept at conducting qualitative research, it can provide a structured approach to understand the processes taken by authors of such work. An overview paper like this may make the methods interesting enough to inspire a new faculty member to learn even more about these useful research methods.

### 5. Tavakol M, Sandars J. Quantitative and qualitative methods in medical education research: AMEE Guide No 90: Part II. *Med Teach*. 2014;36(10):838–48.^17^

#### Summary

This article is the second publication in a two-part series discussing the application of quantitative and qualitative research methodology in medical education.^17^,^18^ While the first article focused more on the importance and differences between the two approaches,^18^ this article provides a thorough overview of the major components of qualitative research.^17^ The authors begin by discussing three common forms of qualitative research: phenomenology (the study of events and occurrences), ethnography (the study of specific cultural groups), and grounded theory (the study of viewpoints and shared meanings). Next, they discuss how to select appropriate populations and how sample size differs from the quantitative approach. Finally, the authors discuss measurement and analysis of the data, emphasizing numerous unique and important features to qualitative assessment.

#### Relevance to Junior Faculty Members

As noted earlier, qualitative methodologies may not be as familiar to researchers as the more traditional quantitative approaches seen in the basic sciences. However, an understanding of qualitative methodologies is very important, as it is particularly relevant within medical education research. Qualitative research provides an opportunity to both discover new theories and to inductively test existing models and theories. This paper provides an overview of the processes involved, as well as how the various components differ from quantitative methods. Readers may find the discussion of sampling, data measurement and analysis particularly valuable as a basis for further reading on the subject, as well as a primer to improve their understanding and critical appraisal when reviewing other qualitative studies.

#### Considerations for Faculty Developers

Rather than relying on hunches, medical educators must make decisions based on the best available evidence. Tavakol’s is the second paper in this series to focus on qualitative methods, highlighting the importance of qualitative methods for consumers of the medical education literature. Faculty members may be less familiar with qualitative methods, since quantitative methods dominate traditional medical education curricula. Qualitative methods facilitate researchers in the “discovery” of medical education theory or in clarifying mechanisms for *why* phenomena occur.^19^ Therefore, educators must be adept in this methodology to conduct and to understand studies in medical education. Faculty development for medical educators must include instruction or mentorship in many of the methodologies discussed in Tavakol’s overview.

## LIMITATIONS

As with our previous papers, we did not design this study to be an exhaustive, systematic search of the literature. We attempted to seek assistance with finding more papers by using expert consultation, which yielded some important recommended papers. Considering the depth and breadth of our final list, we feel that by using these adjunctive methods we have overcome the limitations of our unstructured collection of papers. Additionally, we used a mix of junior clinician educators and experts in the modified Delphi analysis. While the input from junior educators is valuable from an end-user perspective, it is possible that results may have differed if only experts had been used.

## CONCLUSION

We present five key papers addressing research study design with discussions and applications for junior faculty members and faculty developers. These papers provide a basis from which junior faculty members might build upon for designing and analyzing studies.

## Figures and Tables

**Table:** The complete list of study design literature collected by the authorship team.

Citation	Round 1 initial mean scores (SD) max score 7	Round 2 % of raters that endorsed this paper	Round 3 % of raters that endorsed paper in last round	Top 5 papers
Bordage G, Dawson B. Experimental study design and grant writing in eight steps and 28 questions. *Med Educ*. 2003;37(4):376–385.^9^	6.4 (1.1)	87.5%	100%	1
Crites GE, Gaines JK, Cottrell S, et al. Medical education scholarship: An introductory guide: AMEE Guide No. 89. *Med Teach*. 2014;36(8):657–74.^11^	5.5 (0.9)	87.5%	100%	2
Yarris LM, Deiorio NM. Education research: a primer for educators in emergency medicine. *Acad Emerg Med*. 2011;18 Suppl 2:S27–35.^13^	5.6 (1.2)	87.5%	87.5%	3
Ramani S, Mann K. Introducing medical educators to qualitative study design: twelve tips from inception to completion. *Med Teach*. 2016;38(5):456–63.^16^	5.5 (1.4)	75%	75%	4
Tavakol M, Sandars J. Quantitative and qualitative methods in medical education research: AMEE Guide No 90: Part II. *Med Teach*. 2014;36(10):838–48.^17^	5.8 (1.3)	75%	62.5%	5
Dine CJ, Shea JA, Kogan JR. Generating good research questions in health professions education. *Acad Med*. 2016 Oct 4. [Epub ahead of print].^20^	5.6 (1.3)	62.5%	25%	
Tavakol M, Sandars J. Quantitative and qualitative methods in medical education research: AMEE Guide No 90: Part I. *Med Teach*. 2014;36(9):746–56.^18^	5.6 (1.2)	50%	25%	
Artino AR Jr, La Rochelle JS, Dezee KJ, et al. Developing questionnaires for educational research: AMEE Guide No. 87. *Med Teach*. 2014;36(6):463–74.^21^	5.4 (0.9)	62.5%	12.5%	
Watling CJ, Lingard L. Grounded theory in medical education research: AMEE Guide No. 70. *Med Teach*. 2012;34(10):850–61.^22^	5.0 (1.2)	12.5%	12.5%	
Bordage G, Lineberry M, Yudkowsky R. Conceptual frameworks to guide research and development (R&D) in health professions education. *Acad Med*. 2016 Sep 20. [Epub ahead of print].^23^	4.8 (1.0)	25%	12.5%	
O’Brien BC, Ruddick VJ, Young JQ. Generating research questions appropriate for qualitative studies in health professions education. *Acad Med*. 2016 Oct 4. [Epub ahead of print]^24^	5.5 (1.2)	25%	0%	
Bordage G. Conceptual frameworks to illuminate and magnify. *Med Educ*. 2009;43(4):312–9.^25^	5.1 (1.5)	25%	0%	
Chen HC, Teherani A. common qualitative methodologies and research designs in health professions education. *Acad Med*. 2016 Sep 20. [Epub ahead of print]^26^	5.0 (1.3)	12.5%	0%	
Bhanji F, Cheng A, Frank JR, et al. Education scholarship in emergency medicine part 3: a “how-to” guide. *CJEM*. 2014;16 Suppl 1:S13–8.^27^	4.9 (1.8)	62.5%	0%	
Sharma R, Gordon M, Dharamsi S, et al. Systematic reviews in medical education: a practical approach: AMEE guide 94. *Med Teach*. 2015;37(2):108–24.^28^	4.9 (0.6)	37.5%	0%	
O’Brien BC, Harris IB, Beckman TJ, et al. Standards for reporting qualitative research: a synthesis of recommendations. *Acad Med*. 2014;89(9):1245–51.^29^	4.6 (1.9)	25%	0%	
Sullivan GM, Sargeant J. Qualities of qualitative research: part I. *J Grad Med Educ*. 2011;3(4):449–52.^30^	4.6 (1.2)	25%	0%	
Paradis E. The tools of the qualitative research trade. *Acad Med*. 2016 Sep 20. [Epub ahead of print]^31^	4.5 (0.9)	25%	0%	
Artino AR Jr, Durning SJ, Creel AH. AM last page. Reliability and validity in educational measurement. *Acad Med*. 2010;85(9):1545.^32^	4.5 (1.4)	0%	0%	
Sargeant J. Qualitative research part II: participants, analysis, and quality assurance. *J Grad Med Educ*. 2012;4(1):1–3.^33^	4.3 (1.2)	37.5%	0%	
Bergman E, de Feijter J, Frambach J, et al. AM last page: A guide to research paradigms relevant to medical education. *Acad Med*. 2012;87(4):545.^34^	4.3 (1.5)	25%	0%	
Cook DA, Beckman TJ, Bordage G. Quality of reporting of experimental studies in medical education: a systematic review. *Med Educ*. 2007;41(8):737–45.^35^	4.3 (1.5)	12.5%	0%	
Dicicco-Bloom B, Crabtree BF. The qualitative research interview. *Med Educ*. 2006;40(4):314–21.^36^	4.0 (1.1)	0%	0%	
Cook DA, Bordage G, Schmidt HG. Description, justification and clarification: a framework for classifying the purposes of research in medical education. *Med Educ*. 2008;42(2):128–33.^19^	4.0 (1.2)	0%	0%	
Blanchard RD, Artino AR Jr, Visintainer PF. Applying clinical research skills to conduct education research: important recommendations for success. *J Grad Med Educ*. 2014;6(4):619–22.^37^	3.8 (1.7)	0%	0%	
Kuper A, Lingard L, Levinson W. Critically appraising qualitative research. *BMJ*. 2008;337:a1035.^38^	3.6 (1.1)	12.5%	0%	
Phillips AW, Friedman BT, Durning SJ. How to calculate a survey response rate: best practices. *Acad Med*. 2016 Sep 20. [Epub ahead of print]^39^	3.1 (0.8)	0%	0%	
Ahmed R, Farooq A, Storie D, et al. Building capacity for education research among clinical educators in the health professions: A BEME (Best Evidence Medical Education) Systematic Review of the outcomes of interventions: BEME Guide No. 34. *Med Teach*. 2016;38(2):123–36.^40^	3.0 (1.4)	12.5%	0%	
Azer SA. The top-cited articles in medical education: a bibliometric analysis. *Acad Med*. 2015;90(8):1147–61.^41^	2.0 (0.8)	0%	0%	

*SD*, standard deviation.
